# A Randomized, Controlled Study to Investigate How Bovine Colostrum Fortification of Human Milk Affects Bowel Habits in Preterm Infants (FortiColos Study)

**DOI:** 10.3390/nu14224756

**Published:** 2022-11-10

**Authors:** Susanne Soendergaard Kappel, Per Torp Sangild, Agnethe May Ahnfeldt, Valdis Jóhannsdóttir, Line Juul Soernsen, Lene Boejgaard Bak, Christel Friborg, Sören Möller, Gitte Zachariassen, Lise Aunsholt

**Affiliations:** 1 Comparative Pediatrics and Nutrition, Department of Veterinary and Animal Sciences, Faculty of Health and Medical Sciences, University of Copenhagen, 1870 Copenhagen, Denmark; 2 Department of Neonatology, Copenhagen University Hospital Rigshospitalet, 2100 Copenhagen, Denmark; 3 Hans Christian Andersen Children’s Hospital, Department of Neonatology, Odense University Hospital, 5000 Odense, Denmark; 4 Department of Neonatology, Aarhus University Hospital, 8200 Aarhus, Denmark; 5 Open Patient Data Explorative Network (OPEN), Department of Clinical Research, Odense University Hospital, 5000 Odense, Denmark; 6 Department of Clinical Research, University of Southern, 5000 Odense, Denmark; 7 Department of Clinical Medicine, University of Copenhagen, 2200 Copenhagen, Denmark

**Keywords:** nutrition, preterm infant, fortifier, bowel habits, laxative

## Abstract

Background: Human milk does not meet the nutritional needs to support optimal growth of very preterm infants during the first weeks of life. Nutrient fortifiers are therefore added to human milk, though these products are suspected to increase gut dysmotility. The objective was to evaluate whether fortification with bovine colostrum (BC) improves bowel habits compared to a conventional fortifier (CF) in very preterm infants. Methods: In an unblinded, randomized study, 242 preterm infants (26–31 weeks of gestation) were randomized to receive BC (BC, Biofiber Damino, Gesten, Denmark) or CF (FM85 PreNAN, Nestlé, Vevey, Switzerland) as a fortifier. Stools (Amsterdam Stool Scale), bowel gas restlessness, stomach appearance score, volume, and frequency of gastric residuals were recorded before each meal until 35 weeks post-menstrual age. Results: As intake of fortifiers increased, stools became harder in both groups (*p* < 0.01) though less in BC infants (*p* < 0.05). The incidence of bowel gas restlessness increased with laxative treatments and days of fortification in both groups (*p* < 0.01), but laxatives were prescribed later in BC infants (*p* < 0.01). With advancing age, stomach appearance scores improved, but more so in BC infants (*p* < 0.01). Conclusions: Although there are limitations, a minimally processed, bioactive milk product such as BC induced similar or slightly improved bowel habits in preterm infants.

## 1. Introduction

In very preterm infants with an immature gastrointestinal tract (GIT), a successful transition from parenteral to enteral feeding requires the gradual maturation of digestive functions and GIT motility that depend on both endogenous control (e.g., hormonal and nervous regulation) and exogenous factors (e.g., diet and gut microbes) [1]. In general, symptoms related to GIT motility may be divided into bowel habits and feeding intolerance (FI). In this context, infant bowel habits can be defined by parameters such as stool frequency, consistency, volume, and color, but only a few studies have described the factors that influence all these parameters in hospitalized, very preterm infants [2,3]. In clinical practice, FI can be defined as a gastric residual volume of more than 50% of the last feed, abdominal distention, or emesis, or both, and disruption of the patient’s feeding plan [4]. Symptoms of functional GIT disorders, such as constipation and bowel gas restlessness (redness of the face, abdominal bloating, and grunting), often appear during the first weeks of life in very preterm infants [5]. The symptoms are distressing in infants and among parents and healthcare professionals, and diagnostic tools to help distinguish harmless symptoms from more serious disorders are lacking. The distress may or may not involve pain [6]. Symptoms of FI may lead to less increase in enteral nutrition, prolonged use of parenteral nutrition, and poor growth [7,8]. Symptoms of FI decrease with increasing postnatal age, enteral feeding volume, and bacterial colonization [9,10]. Due to the uncertain relation to other morbidities (e.g., necrotizing enterocolitis (NEC) and gastrointestinal diseases), FI often leads to a cessation of enteral feeding, potentially inducing growth restriction and impaired development of critical organs, including the brain [11].

During the neonatal period, the mother’s own milk (MOM) is the optimal source of enteral nutrition in very preterm infants [12]. If MOM is not available or an insufficient volume is achieved, donor human milk (DHM) is considered the second-best choice [13], but neither MOM nor DHM contains enough protein and minerals to support optimal growth in very preterm infants [14]. Therefore, nutrient fortifiers are added to MOM and/or DHM. These are typically manufactured from cow’s milk protein, with added maltodextrin, vegetable oils, and minerals. However, these products have been suspected to negatively affect FI and gut dysmotility [15], and a few cases of bowel obstruction after the intake of fortification have been reported [16,17]. The optimal fortifier product is still under debate. Possibly, products based exclusively on human milk are superior to products based on bovine milk, but their production incurs ethical, practical, and technical challenges, as well as high costs [18]. 

Across all mammals, the first milk after parturition, colostrum, is a particularly rich source of nutrients and contains GIT-protective bioactive factors [19]. For bovine colostrum (BC), the total levels of protein (casein, whey proteins and immunoglobulins) are six times higher than in human colostrum and ten times higher than in mature human milk [20]. Likewise, concentrations of bioactive components, as well as lactoferrin and many growth factors, are high [21]. In preterm piglets, used as a model for preterm infants, early feeding with BC improves nutrient intake, growth, and NEC resistance, compared with infant formula products [22,23]. When used as a fortifier to human milk, preterm piglets show improved gut maturation, growth, and infection resistance relative to commercially available fortifiers [24]. Based on these findings, we hypothesized that a fortifier based on intact BC is well tolerated by very preterm infants, and improves bowel habits in very preterm infants, compared with a conventional fortifier (CF). We therefore evaluated a series of clinical parameters of bowel habits and feeding tolerance in very preterm infants, randomized to human milk fortified with either BC or CF.

## 2. Materials and Methods

The study was performed as a pragmatic, unblinded multi-center, randomized controlled study conducted from December 2017 to November 2020 at eight neonatal units in Denmark (part of the NEOCOL program, Innovation Fund Denmark), according to a previously published protocol (NCT03537365 Clin.trial.gov) [25]. The study was conducted to ensure similar growth but was designed to investigate NEC and late-onset sepsis as the primary outcome. The study conducted was not able to investigate the latter outcomes. The investigation of FI was a secondary outcome, superficially described in the protocol paper [25]. These data are to be reported separately. Infants were eligible if gestational age at birth was between 26 + 0 and 30 + 6 (weeks + days), and nutrient fortification was needed to achieve optimal growth. The intervention was expected to proceed until post-menstrual age 34 + 6 weeks at one of the participating units (Aarhus University Hospital, Aarhus; Herlev Hospital, Herlev; Hospital Soenderjylland, Aabenraa; Hvidovre Hospital, Hvidovre; Hospital Lillebaelt, Kolding; North Zealand Hospital, Hilleroed; Odense University Hospital, Odense; Rigshospitalet University Hospital, Copenhagen). Exclusion criteria were major congenital anomalies and birth defects, formula feeding or gastrointestinal surgery before the start of fortification. Allergic disposition was not included as an exclusion criterion, since previous studies in comparable cohorts did not find an increased risk of milk protein allergy. The Regional Scientific Ethical Committee approved the study of Southern Denmark (S-20170095) and the Danish Data Protection Agency (17/33672). An independent data safety monitoring board followed the study.

### 2.1. Recruitment and Randomization of Participants

Physicians and nurses selected eligible very preterm infants during their first week of life. After obtaining oral and written informed consent from both parents, infants were randomized to a conventional bovine milk-based fortifier (CF group; FM85, PreNAN, Nestlé, Vevey, Switzerland) or BC as the fortifier (BC group; BC, Biofiber Damino, Gesten, Denmark). Computer-generated randomizations were performed using a secure website (REDCap) hosted by the Region of Southern Denmark [26]. The randomization sequences were generated with a 1:1 allocation, using random block sizes of 4–6, and stratified as small for gestational age (SGA, defined as birth weight less than or equal to 2.0 standard deviations below the mean from the growth reference used in Denmark [27]). In the case of multiple births, all infants were allocated to the same treatment group. The study was performed unblinded, as both clinical personnel and parents were able to distinguish human milk mixed with BC or CF based on differences in color and consistency.

### 2.2. Intervention

In line with pragmatics, each participating site followed its local guideline for enteral feeding. MOM and/or DHM is the standard enteral feeding for infants with a gestational age (GA) below 32 + 0, where DHM is only used if the volume of MOM is insufficient to cover the total daily volume requirement. The fortification of human milk was initiated at an enteral feeding volume of 100 mL/kg/day and before 140 mL/kg/day, unless blood urea nitrogen (BUN) values were above 5.0 mmol/L. In such cases, the start of fortification was postponed until BUN values were below 5.0 mmol/L. The fortification was continued at all sites until GA week 34 + 6.

The composition of the powdered fortifier products is shown in Table 1. In both groups, the fortification was initiated with 1.0 g of fortifier powder added to 100 mL of human milk. Because the protein concentration was higher in BC versus CF powder (0.50 g/1 g BC powder versus 0.35 g/1 g CF powder), the amount of powder required to obtain the same protein fortification level was different. Thus, the maximum level of fortification was 2.8 g/100 mL in the BC group and 4.0 g/100 mL in the CF group. Individualized fortification was standard procedure at all sites.

A standard operation procedure (SOP) described how to dissolve BC into cold human milk using a handheld milk mixer (15 s) or manually stirring after moderate heating of the MOM and/or DHM. The CF was dissolved into cold human milk by gentle shaking. Infants in both groups were supplemented with multi-vitamins, vitamin D, iron, zinc, phosphorus, and calcium, according to national guidelines [28]. BC was manufactured without extra added vitamins and minerals, and subsequently, infants randomized to BC were, according to an SOP, recommended to be given more supplements of phosphate, vitamin, and minerals than infants randomized to CF. All other aspects of feeding and care followed routine clinical practices at each unit. The intervention stopped at 34 + 6 weeks post-menstrual age, or earlier if the infant was transferred to a non-participating unit, was discharged to an early discharge program, or if formula feeding was introduced.

### 2.3. Gastrointestinal Data Collection and Management

Data regarding FI, bowel habits, and pain were recorded using a pre-designed case report form (CRF). The CRF was developed based on an existing clinical guideline for gastric residual (GR), the validated Amsterdam Stool Scale, and the ComfortNeo pain scale [29,30,31]. The quality and clarity of the CRF were ensured partly based on responses from ten neonatal nurses prior to the start of the study. The final CRF (Supplementary Materials Figure S1) included observations of GR volume, GR color (milk, lemon, mustard, wasabi, lime, avocado, and spinach [30]), feeding volume (planned and actual), type of milk (MOM, and/or DHM), type and amount of fortification (g/100 mL) and stomach appearance (inconspicuous, air, visible blood vessels, visible bowel loops, lustrous and discoloration), stool frequency (number per day), volume (smear, up to 25%, 25–50%, >50%), color (I–VI) and consistency (watery, soft, formed, hard) [29]. Finally, infants were evaluated with a ComfortNeo score [31] and an evaluation of the presence or absence of bowel gas restlessness. During the intervention period, the CRF was filled out by nurses or parents at each meal. Episodes of feeding intolerance, defined as a GR above 50% of the previous meal, the appearance of a GR (yes/no), reductions in the volume of enteral nutrition, stomach appearance, and ComfortNeo scores > 14/day were recorded. Baseline clinical characteristics and medication were extracted from the electronic medical records. All data were entered in the online database (REDCap) [26].

### 2.4. Statistics

All statistical analyses were performed using the statistical software R (version 4.0.0, R Foundation for Statistical Computing, Vienna, Austria). Only infants fortified for at least 14 days were included in the analyses, and individuals diagnosed with NEC or ileus were excluded to avoid possible interacting effects of these gut morbidities. For baseline characteristics, demographic factors and clinical categorical variables were summarized and compared between groups using the chi-square test. Normally distributed variables were reported as mean with standard deviation (SD) and compared using Student’s *t*-test. Log-rank tests were applied to compare time-to-event data (e.g., time to first fortified meal). Infant data were recorded for a variable number of days due to differences in the length of intervention across infants. Complete CRF sheets were available for 16–55 days (mean 34 days). To avoid excessive missing values, only data recorded until day 34 of the intervention were included. The differences between groups were evaluated shortly after the start of fortification (from day 1 to 7 days) to assess if the introduction of fortifiers induced short-term side effects. Furthermore, analysis was performed across the entire fortification period (from day 1 to 34 days) to assess the long-term effects of the intervention. Numeric outcomes were analyzed using mixed effects linear regression for repeated data, including a random intercept for each infant. Dichotomous outcomes were analyzed using a mixed effects logistic regression model, and ordinal data using mixed effect ordinal logistic regression models. When analyzing stool color, these were divided into normal (score I–IV) and abnormal (score V–VI) colors and analyzed as dichotomous outcome. Results were presented as regression coefficients and odds ratio (OR) with 95% confidence intervals (95%CIs). Survival analyses, using the Cox regression model, were used to analyze time-to-event outcomes. All models were adjusted for a predefined list of covariates. These consisted of GA at birth, postmenstrual age, dietary protein from fortifier (g per (pr.) meal), and geographical area (East or West Denmark). While performing the study, BC was more often randomized in East compared to West Denmark, and we found higher amounts of MOM and higher infant growth rates in West compared to East Denmark (data published elsewhere), hence the geographical area was added as a covariate. The use of laxatives and antibiotics at each meal was included as covariates when assessing GIT motility. Both may induce changes in stool consistency and frequency, and antibiotics may indicate illness with effects on GIT motility. Furthermore, nasal-CPAP (N-CPAP) treatment (yes/no) was included as a covariate in the analysis of stomach appearance, since it is common knowledge that N-CPAP may affect infant abdominal appearance. In addition, when assessing GR, the volume of the prescribed previous meal was included as a covariate since the definition of FI includes aspirates above 50% of the previous meal. Covariates were included in all analyses to avoid overfitting by data-driven covariate selection [32]. Missing values in numeric variables (e.g., planned and actual number of meals and meal volumes) were handled using interpolation. A *p*-value < 0.05 was considered statistically significant, and *p*-values < 0.1 were considered to indicate the tendency of an effect [33].

## 3. Results

### 3.1. Inclusion and Demographic Variables

**Figure 1:**
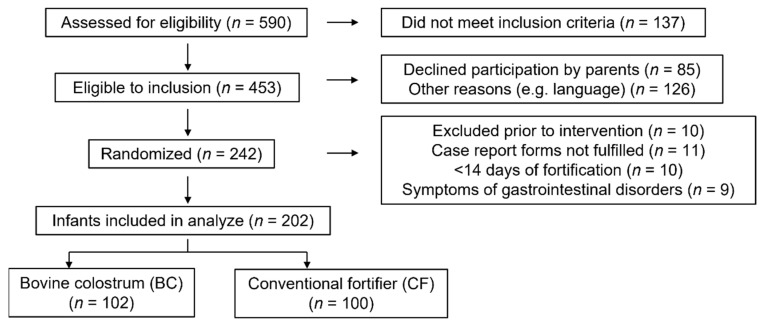
Consort of flow of participants.

A diagram of the recruitment of the 242 randomized very preterm infants is shown in Figure 1. Ten infants were excluded before the start of intervention due to the withdrawal of parental consent, transfer to a non-participating hospital unit or death of the infant. Nine infants were diagnosed with NEC (BC: 3/115 vs. CF: 5/117, *p* = 0.72) or ileus (CF: 1/117). Eleven infants were excluded because the parents declined to fill out the observation forms. Ten randomized infants did not need fortification (due to adequate growth according to the local guideline) or had less than 14 days of fortification. Among the remaining 202 very preterm infants, *n* = 102 received BC, and *n* = 100 received CF as fortifiers.

At baseline, no differences were observed for demographic variables and clinical characteristics, except that BC infants started fortification one day earlier than CF infants (*p* < 0.05, Table 2).

**Figure 2:**
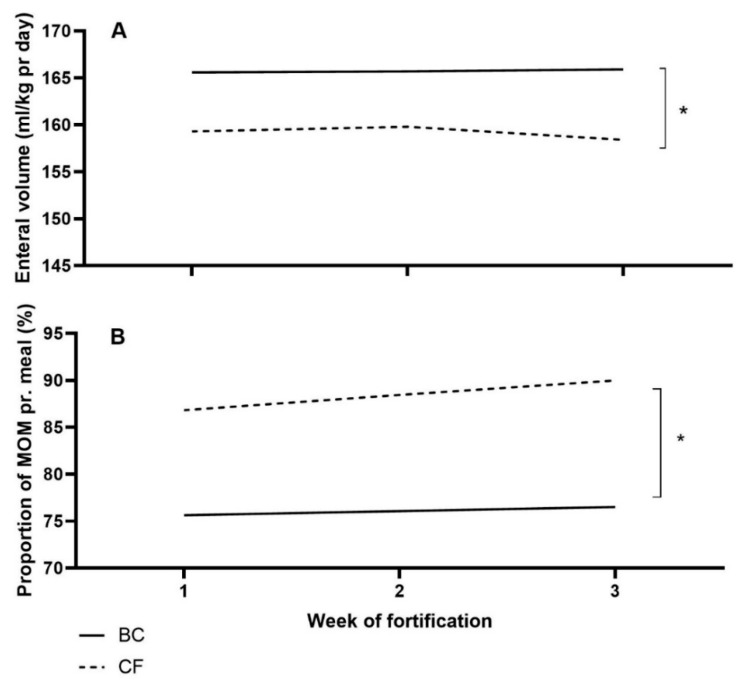
Mean volume of enteral nutrition per kg pr. day (**A**), and the proportion of the mother’s own milk (MOM) per meal (**B**) for Bovine colostrum (BC) and Conventional fortifier (CF). pr.: per. * *p* < 0.05.

During the intervention, human milk (MOM and DHM) was fortified with a similar amount of protein in grams in the two groups, but BC infants received a higher total volume of enteral nutrition pr. day (*p* < 0.01, Figure 2A). Finally, BC infants received a lower proportion of MOM than CF infants (*p* < 0.05, Figure 2B).

### 3.2. Bowel Habits

**Figure 3:**
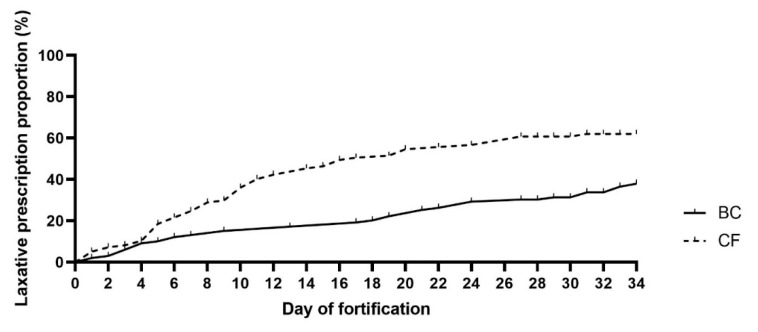
Time of initiation of prescribed laxatives. Bovine colostrum (BC) and Conventional fortifier (CF). Based on Survival analyses, using the Cox regression model.

Defecation frequency was similar between BC and CF infants during the first week of fortification (*p* = 0.3), but analyzed across the entire observation period, BC infants tended to defecate more frequently (OR: 1.01, 95%CI (1.00, 1.03), *p* = 0.07). There were no differences between the two groups in stool volume, and stool consistency was similar between BC and CF infants during the first week of intervention (*p* = 0.24). However, across the entire intervention period, the stool consistency score was lower (softer stool) in infants fortified with BC (OR: 0.99, 95%CI (0.986, 0.996), *p* < 0.01). During the first week of fortification, the risk of having an abnormal stool color was lower in the BC infants (OR: 0.74, 95%CI (0.59, 0.93), *p* < 0.05). However, the groups showed similar stool color across the entire observation period (*p* = 0.17). The two groups had similar events with bowel gas restlessness (0–7 days: *p* = 0.11 and 0–34 days: *p* = 0.19, respectively), but laxatives were prescribed later in the BC versus CF group (*p* < 0.01, Figure 3).

### 3.3. Feeding Intolerance

Feeding tube aspiration was performed in 77% of the feeds. The incidence of GR (>50% of the last meal retained in the stomach) was similar between groups, both during the first week of fortification and across the entire intervention period. The appearance of GR (disregarding GR volume) was similar between groups throughout the entire intervention period. Across the entire intervention period, stomach appearance scores were lower in the BC compared to the CF group (OR: 0.991, 95%CI (0.987, 0.994), *p* < 0.01). Finally, there was no difference between groups in obtaining the prescribed volume of milk per meal (*p* = 0.28), and there were no differences in the ComfortNeo score (data not shown) between the two groups at any time during the intervention (0–7 days: *p* = 0.34, 0–34 days: *p* = 0.73).

### 3.4. Results of the Co-Variant Analysis

In both groups, defecation frequency increased during the first week after the initiation of fortification as the amount of supplied protein was increased (Table 3). Furthermore, analyzed across the entire intervention period, infants born SGA had lower defecation frequency than non-SGA infants (Table 3). The volume of stools per observation was larger in infants receiving laxatives in the first week of fortification and tended to increase with an increased amount of added fortifier (Table 3). In the case of antibiotic prescription, the volume of stools decreased, as analyzed across the entire observation period (Table 3). As the intake of protein from fortifiers increased, stool consistency in both groups became harder. In addition, infants receiving laxatives across the intervention period had higher stool consistency scores (harder stool, Table 3). During the first week of fortification, the stool color was more abnormal in SGA infants across groups and became more normal across groups with increasing postnatal age (Table 3). Furthermore, during the entire observation period, the stool color became more abnormal in all included infants in the case of the use of antibiotics and in infants born SGA (Table 3), whereas the stool color became more normal as intake of protein from fortifiers increased and after prescribed laxatives (Table 3). In both groups, the events with bowel gas restlessness during the first week of fortification increased with advanced GA at birth, postnatal age, and when infants received laxatives (Table 3). Infants receiving antibiotics in the first week of fortification had fewer cases of bowel gas restlessness (Table 3).

With an increasing volume of meals, there was a decreased risk of a GR both during the first week of fortification and across the entire observation period (Table 3). However, if antibiotics were prescribed at any time during intervention, the incidence of GR increased (Table 3). During the first week, an increasing amount of fortification was correlated with more frequent GR appearance in both groups (Table 3). However, for infants born SGA, there was a decreasing frequency of GR appearance in the first week of fortification (Table 3). Subsequently, across the entire observation period, there was decreased incidence of GR, despite an increase in the volume of meals (Table 3). However, if antibiotics were prescribed at any time during the intervention, the incidence of having GR increased (Table 3). The stomach appearance scores decreased with advancing days of fortification and higher GA at birth (Table 3). Conversely, these scores increased with an increasing amount of protein from fortifiers, postnatal age, SGA at birth, treatment with N-CPAP, laxative, or antibiotics, both in the first week and across the entire period of fortification (Table 3).

## 4. Discussion

Human milk is considered the best source of nutrition in very preterm infants, but due to its insufficient content of macronutrients, fortification is often needed [12,14]. We investigated the response to a novel fortifier based on an intact BC product in very preterm infants because conventional fortifiers are suspected to negatively affect bowel habits and FI [16,17]. BC, as a fortifier, was in our study superior compared to a conventional fortifier regarding stool consistency. Furthermore, the BC group received laxatives later than CF infants, and the qualitative clinical evaluation of the abdomen was similar or improved. We demonstrated that the quality of fortification products influences bowel habits and FI and that a novel product based on BC has no apparent negative effects but a marginally positive effect on some parameters of bowel habits. We did not include a group of infants that were non-fortified; hence were unable to demonstrate the specific effects of BC fortification itself on bowel habits and FI parameters. Based on a long series of assessments of bowel movement and function, analyzed against a range of clinically-relevant co-variates, our study also provides baseline data of clinical evaluations by nurses, physicians, and parents that may help to prevent adverse effects of feeding regimens for very preterm infants.

Processed formula products reduce defecation frequency in preterm infants relative to human milk [34]. We found a tendency for more frequent defecation when BC was used as a fortifier, indicating that BC may prevent constipation. The effect may relate to the high amount of immunoglobulin G and other proteins in the BC product [35], which may pass through the immature gut partly undigested [36], thereby contributing to motility and having a laxative effect with softer stools. The gut luminal effects of intact immunoglobulins, either human or bovine, may protect against GIT infections in infants [35], but the incomplete digestion of protein may also negatively affect the total amino acid supply. Suspected reduced digestibility of BC versus CF protein, more DHM (with less protein than MOM), and an associated poorer growth observed in the neonatal intensive care unit (NICU) may explain why the feeding volume was adjusted to a higher level in BC compared to CF infants. According to our protocol and SOPs, clinical personnel were free to adjust nutrition and fortification according to standard practices and growth targets accepted by each unit. In ongoing studies, we are investigating whether dietary protein from BC, specifically immunoglobulins, is less digestible in preterm infants relative to the hydrolyzed whey protein supplied by the CF fortifier (FM85, Nestle).

In clinical settings, the fortification of human milk has been shown to contribute to bowel obstruction [16], and in some studies, very preterm infants given nutrient fortification were more often treated with laxatives than non-fortified infants [37]. Often, the prescription of laxatives is empiric rather than evidence-based [38], and few diagnostic tools for constipation in preterm infants are available [39]. Evaluation of stool consistency, amount, and color by the Amsterdam Stool Scale (ASS) in this study gave a uniform description of stools at all participating units. Nevertheless, this scale lacks guidance on when laxative needs to be prescribed [29]. The ROME IV criteria describe functional constipation only for infants and children, not preterm infants [40]. In the present study, infants fortified with CF were more often observed with hard stools relative to BC infants, and they received laxative treatment earlier than infants who received BC. The earlier treatment could be explained by clinical personnel expecting more functional constipation in infants receiving CF, but one could speculate whether the differences in ion content in the two products may induce harder stools earlier and, as such, have influenced the results. The use of laxatives may increase intestinal fermentation [41] and thereby cause a state of ‘bowel gas restlessness’, as confirmed by our results. It is challenging to discriminate bowel gas restlessness from infant dyschezia since the latter definition includes strain and crying for a minimum of 10 min before successful or unsuccessful passage of soft stools [38]. Furthermore, discrimination between dyschezia and constipation is difficult and may lead to more use of laxatives; collectively, these are clinical signs of immature food passage and gut motility [40].

When defined as a combination of large GR volumes, together with abdominal distention or emesis, feeding intolerance (FI) may reflect gut dysmotility due to immaturity [4]. FI is often present in preterm infants and may lead to a reduction or pause in enteral nutrition, with a subsequent negative effect on growth velocity [42]. In preterm infants, previous studies have shown an increase in GR following conventional human milk fortification [43]. Conversely, GR increased in preterm piglets fortified with BC compared to CF, possibly reflecting excessive casein clotting in the stomach that would only be relevant at high levels of BC fortification [44]. In this study, we found no difference in GR between groups, indicating that the higher content of casein in BC [20] did not induce any risk of increased GR. However, the exact volume of GR is often uncertain as gastric aspiration depends on factors such as placement and size of the feeding tube, infant body position and viscosity of content [45,46]. Statistically, the presence of GR (regardless of volume) had no negative effect on the following feeding volume, indicating that neither BC nor CF increased FI. Infants fortified with BC had a lower stomach appearance score, potentially reflecting a more mature gut, including improved motility and digestive function, as suggested by preterm piglets fortified with BC [47,48].

Clinically relevant covariates were chosen and considered while analyzing data. We found that GA at birth influenced bowel gas restlessness; the older the infant, the more bowel gas restlessness. One could speculate whether this is due to difficulties in the interpretation of symptoms in a very immature infant compared to a less immature infant, more than the age itself, since bowel gas restlessness would have been expected to be less pronounced with improved motility. Furthermore, we found that the use of antibiotics resulted in low stool volume, quite the opposite of what would have been expected since daily clinical observations more often indicate diarrhea-like stools during antibiotic courses. One could speculate whether this phenomenon is due to reduced nutritional intake during critical illness and paralytic ileus in cases with severe late-onset sepsis. This study was designed as an open-label, randomized non-powered study, with potential bias from observing personnel. Despite the possible bias, this design was considered acceptable for investigating the use of BC in clinical praxis. The evaluations performed were both objective and subjective, but all observers were trained. Neither parents, nurses, nor doctors had any prior knowledge of BC in preterm infants, but it cannot be excluded that the general concern for adverse effects of CF influenced their opinion. Scales used in the observation form were both validated (ASS) and non-validated (e.g., stomach appearance score, bowel gas restlessness) with some variability among evaluators. Even though we used non-validated scales, we found similar results as previously described by others in terms of the effects of N-CPAP on bowel gas restlessness, indicating that our scales may be reliable [49]. In this study, we included structural observations of bowel habits and FI, which are often lacking in other nutritional studies, where these are either more subjective or simply described. Future studies should consider including interdisciplinary competencies when designing their studies to optimize and further validate our results.

## 5. Conclusions

In conclusion, the fortification of human milk with intact BC, targeting protein recommendations and growth rates, does not negatively affect very preterm infants but may induce softer stools, less adverse abdominal appearance, and less/later treatment with laxatives. In turn, this could improve the infant’s quality of life. It appears safe from a bowel habit perspective to use BC as a novel fortifier for human milk to very preterm infants. Authors should discuss the results and how they can be interpreted from the perspective of previous studies and of the working hypotheses. The findings and their implications should be discussed in the broadest context possible. Future research directions may also be highlighted.

## Figures and Tables

**Table 1:** Nutrient supply of bovine colostrum and the conventional fortifier (FM85 PreNAN) when the maximum fortification reached pr. 100 mL of human milk.

	Bovine Colostrum	FM85 PreNAN
	pr. 2.8 g	pr. 4 g
Energy (kcal)	13	17
Protein (g)	1.4	1.4
Carbohydrate (g)	0.6	1.3
Fat (g)	0.6	0.72
Calcium (mg)	25.8	76
Phosphorus (mg)	22.7	44
Zink (mg)	0.2	0.96
Iron (mg)	0	1.8
Vitamin D3 (µg)	0	3.5
Vitamin A (µg)	27.8	333
Vitamin E (mg)	0.05	3.8
Vitamin C (mg)	0	19

pr.: per.

**Table 2:** Baseline characteristics of included infants (percentage, median min-max or means ± SD).

	Bovine Colostrum	FM85 PreNAN	*p*-Value
*N*	102	100	
Antenatal steroid *n* = 201 (%)	96	93	0.54
Cesarean section (%) *n* = 202	69	74	0.49
Multiple births (%) *n* = 202	29	34	0.69
Gender (% males) *n* = 202	62	67	0.33
Gestational age at birth (weeks), median (min-max) *n* = 202	28.9 (26–30.9)	28.6 (26–30.9)	0.33
Small for gestational age (%) *n* = 202	23.5	22.0	0.93
Birth weight (g) *n* = 202	1183 ± 337	1157 ± 322	0.58
Apgar score, 5 min, median (min-max) *n* = 191	9 (2–10)	10 (4–10)	1.00
Mechanical ventilation (%) *n* = 202	4.9	8.0	0.54
Mechanical ventilation (days) *n* = 41	23 ± 19	21 ± 15	0.83
Continuous positive airway pressure (%) *n* = 202	82	86	0.61
Continuous positive airway pressure (days) *n* = 143	27 ± 24	31 ± 24	0.29
PMA at start of fortification (weeks), median (min-max) *n* = 202	30.1 (26.9–32.3)	30.1 (27.6–32.1)	0.80
Body weight at the start of fortification (g) *n* = 202	1138 ± 291	1131 ± 280	0.87
Postnatal age at start of fortification (days), median (min-max) *n* = 202	8 (3–17)	9 (4–26)	0.03 *
Feeding volume at start of fortification (mL/kg) *n* = 202	148 ± 20	147 ± 21	0.57
PMA: postmenstrual age			

SD: standard deviation, min: minimum, max: maximum. * *p* < 0.05

**Table 3:** The effect of Co-variates t on bowel habits and feeding intolerance. Numeric outcomes were analyzed using mixed effects linear regression for repeated data, including a random intercept for each infant. Dichotomous outcomes were analyzed using a mixed effects logistic regression model, and ordinal data using mixed effect ordinal logistic regression models.

	Day 0–7			Day 0–34		
	OR	95%CI	*p*-Value	OR	95%CI	*p*-Value
Frequency of defecation						
Advancing days of fortification	0.97	0.92–1.02	ns	0.98	0.95–1.02	ns
Increasing amount of protein	1.44	1.01–2.06	<0.05	1.00	0.81–1.24	ns
GA at birth	1.04	0.96–1.12	ns	1.02	0.95–1.09	ns
Small for gestational age	0.87	0.71–1.07	ns	0.78	0.65–0.94	<0.01
Postnatal age	1.00	0.97–1.04	ns	1.01	0.98–1.04	ns
Receiving laxatives	0.91	0.73–1.13	ns	1.06	0.89–1.25	ns
Receiving antibiotics	1.08	0.83–1.39	ns	1.08	0.90–1.29	ns
Volume of stool						
Advancing days of fortification	1.03	0.99–1.09	ns	1.01	0.99–1.03	ns
Increasing amount of protein	1.20	0.98–1.54	0.09	1.17	1.09–1.24	<0.01
GA at birth	0.95	0.92–1.05	ns	0.99	0.69–1.04	ns
Small for gestational age	0.94	0.78–1.13	ns	0.99	0.92–1.16	ns
Postnatal age	0.99	0.97–1.03	ns	1.00	0.99–1.02	ns
Receiving laxatives	1.42	1.13–1.91	<0.01	0.98	0.92–1.04	ns
Receiving antibiotics	0.87	0.70–1.09	ns	0.87	0.80–0.95	<0.01
Stool consistency						
Advancing days of fortification	1.00	0.96–1.04	ns	0.99	0.96–1.01	ns
Increasing amount of protein	1.11	1.02–1.25	<0.05	1.19	1.12–1.27	<0.01
GA at birth	1.04	0.97–1.12	ns	1.02	0.96–1.08	ns
Small for gestational age	1.03	0.83–1.25	ns	0.99	0.87–1.17	ns
Postnatal age	1.01	0.98–1.04	ns	0.99	0.98–1.02	ns
Receiving laxatives	1.46	1.21–1.73	<0.01	1.10	1.02–1.21	<0.05
Receiving antibiotics	1.00	0.84–1.18	ns	1.01	0.92–1.12	ns
Stool color						
Advancing days of fortification	1.11	0.92–1.35	ns	1.02	0.95–1.10	ns
Increasing amount of protein	0.65	0.34–1.23	ns	0.59	0.37–0.95	<0.05
GA at birth	0.99	0.82–1.19	ns	1.01	0.80–1.27	ns
Small for gestational age	1.72	1.12–2.64	<0.05	1.72	1.16–2.53	<0.01
Postnatal age	0.88	0.80–0.97	<0.01	0.96	0.89–1.03	ns
Receiving laxatives	1.28	0.60–2.33	ns	0.60	0.35–1.02	0.06
Receiving antibiotics	1.48	0.80–2.75	ns	1.68	0.99–2.84	0.05
Bowel gas restlessness						
Advancing days of fortification	0.94	0.82–1.08	ns	0.95	0.88–1.02	ns
Increasing amount of protein	0.93	0.38–2.30	ns	1.26	0.74–2.16	ns
GA at birth	1.31	1.03–1.66	<0.05	1.10	0.92–1.32	ns
Small for gestational age	1.03	0.59–1.79	ns	1.06	0.72–1.55	ns
Postnatal age	1.23	1.12–1.34	<0.01	1.08	1.01–1.16	<0.01
Receiving laxatives	2.74	1.61–4.64	<0.01	2.65	1.86–3.76	<0.01
Receiving antibiotics	0.44	0.21–0.95	<0.05	0.50	0.33–0.75	<0.01
Appearance of GR >50% of last meal						
Advancing days of fortification	1.02	0.75–1.38	ns	0.85	0.73–0.98	<0.05
Increasing amount of protein	0.75	2.16–2.60	ns	0.86	0.34–2.16	ns
GA at birth	1.69	1.09–2.62	<0.05	1.60	1.03–2.50	<0.05
Small for gestational age	0.62	0.22–1.72	ns	0.63	0.22–1.79	ns
Postnatal age	0.99	0.84–1.17	ns	1.00	0.87–1.16	ns
Receiving laxatives	2.21	0.73–6.61	ns	2.01	0.72–5.61	ns
Receiving antibiotics	1.02	0.92–6.17	0.07	2.92	1.18–7.27	<0.05
Volume of meal	0.78	0.69–0.87	<0.01	0.79	0.71–0.89	<0.01
Appearance of GR (regardless of amount)						
Advancing days of fortification	0.96	0.88–1.04	ns	0.99	0.94–1.05	ns
Increasing amount of protein	1.62	1.13–2.32	<0.01	1.05	0.76–1.44	ns
GA at birth	1.00	0.87–1.15	ns	0.75	0.96–1.20	ns
Small for gestational age	0.65	0.46–0.92	<0.05	0.86	0.54–1.04	0.08
Postnatal age	0.97	0.92–1.03	ns	1.00	0.95–1.05	ns
Receiving laxatives	0.94	0.64–1.38	ns	1.11	0.84–1.48	ns
Receiving antibiotics	1.51	1.09–2.09	<0.05	1.41	1.08–1.83	<0.05
Volume of meal	0.98	0.95–1.01	ns	0.97	0.95–0.99	<0.01
Stomach appearance						
Advancing days of fortification	0.88	0.82–0.95	<0.01	0.94	0.89–0.99	<0.05
Increasing amount of protein	1.51	1.24–1.80	<0.01	1.36	1.25–1.42	<0.01
GA at birth	0.75	0.65–0.88	<0.01	0.71	0.63–0.79	<0.01
Small for gestational age	2.75	1.69–4.25	<0.01	2.29	1.62–3.22	<0.01
Postnatal age	1.14	1.06–1.29	<0.01	1.05	1.00–1.10	0.06
Receiving laxatives	1.62	1.34–1.93	<0.01	1.71	1.61–1.85	<0.01
Receiving antibiotics	1.22	1.04–1.43	<0.05	1.39	1.30–1.52	<0.01
N-CPAP treatment	1.50	1.14–1.94	<0.01	1.17	1.06–1.28	<0.01

OR: odds ratio, CI: confidence interval, GA: gestational age, GR: gastric residual, N-CPAP: nosal-Continuous Positive Airway Pressure, ns: not significant.

## Data Availability

Not applicable.

## Supplementary Materials

The following supporting information can be downloaded at: https://www.mdpi.com/article/10.3390/nu14224756/s1, Figure S1: Clips from the observation form.
