# Supplementary material for: A Randomized, Controlled Study to Investigate How Bovine Colostrum Fortification of Human Milk Affects Bowel Habits in Preterm Infants (FortiColos Study)
Source: Nutrients. 2022 Nov 10;14(22):4756. doi: 10.3390/nu14224756 (PMC9696900; doi:10.3390/nu14224756)
Supplement: Supplementary file 1 [file nutrients-14-04756-s001.zip › nutrients-1996160-supplementary.pdf]

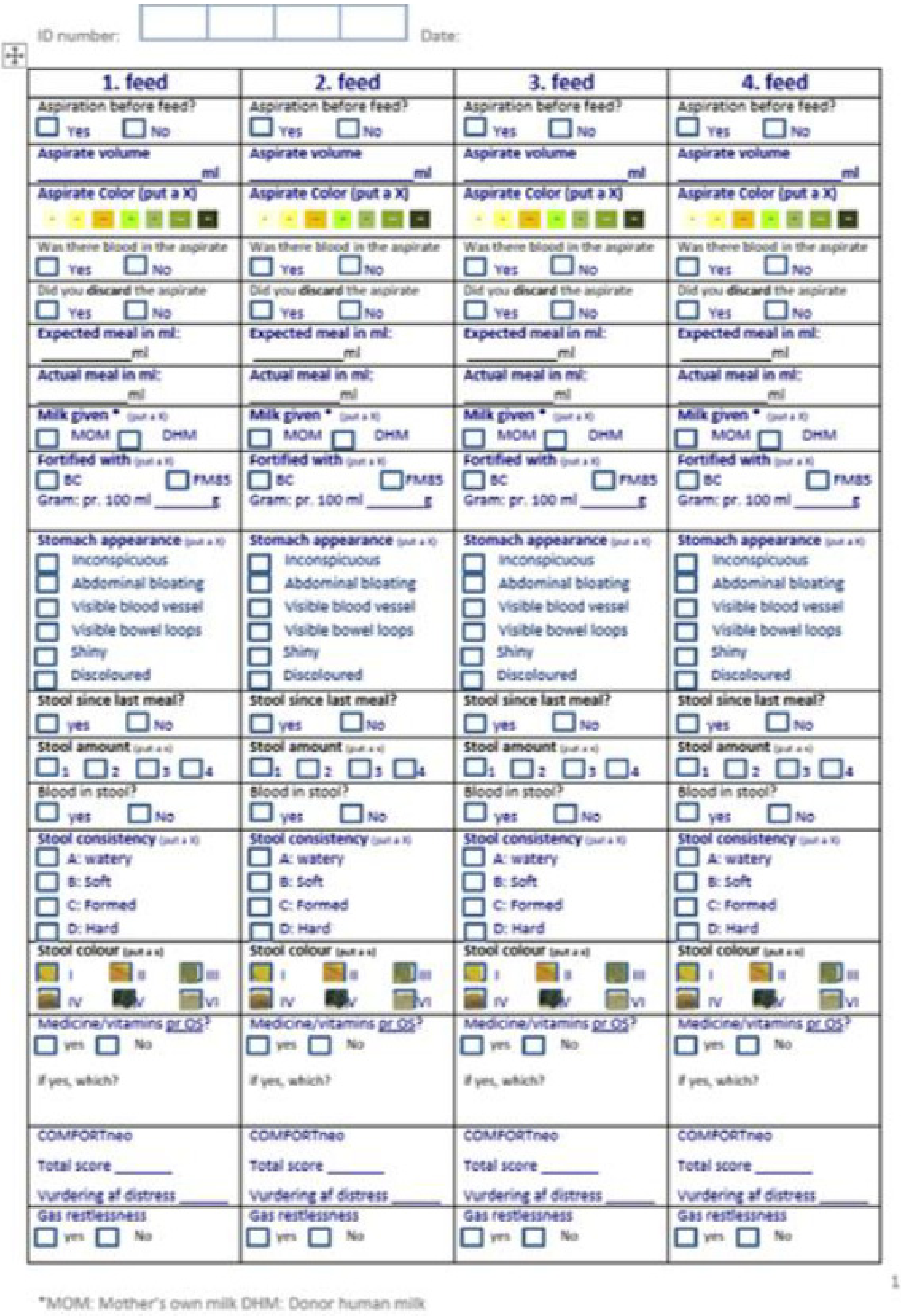

ID number: Date:

1. feed	2. feed	3. feed	4. feed
Aspiration before feed? ☐ Yes ☐ No	Aspiration before feed? ☐ Yes ☐ No	Aspiration before feed? ☐ Yes ☐ No	Aspiration before feed? ☐ Yes ☐ No
Aspirate volume ______ ml	Aspirate volume ______ ml	Aspirate volume ______ ml	Aspirate volume ______ ml
Aspirate Color (put a X)	Aspirate Color (put a X)	Aspirate Color (put a X)	Aspirate Color (put a X)
Was there blood in the aspirate ☐ Yes ☐ No	Was there blood in the aspirate ☐ Yes ☐ No	Was there blood in the aspirate ☐ Yes ☐ No	Was there blood in the aspirate ☐ Yes ☐ No
Did you discard the aspirate ☐ Yes ☐ No	Did you discard the aspirate ☐ Yes ☐ No	Did you discard the aspirate ☐ Yes ☐ No	Did you discard the aspirate ☐ Yes ☐ No
Expected meal in ml: ______ ml	Expected meal in ml: ______ ml	Expected meal in ml: ______ ml	Expected meal in ml: ______ ml
Actual meal in ml: ______ ml	Actual meal in ml: ______ ml	Actual meal in ml: ______ ml	Actual meal in ml: ______ ml
Milk given * (put a X) ☐ MOM ☐ DHM	Milk given * (put a X) ☐ MOM ☐ DHM	Milk given * (put a X) ☐ MOM ☐ DHM	Milk given * (put a X) ☐ MOM ☐ DHM
Fortified with (put a X) ☐ BC ☐ FM85 Gram: pr. 100 ml ______ g	Fortified with (put a X) ☐ BC ☐ FM85 Gram: pr. 100 ml ______ g	Fortified with (put a X) ☐ BC ☐ FM85 Gram: pr. 100 ml ______ g	Fortified with (put a X) ☐ BC ☐ FM85 Gram: pr. 100 ml ______ g
Stomach appearance (put a X) ☐ Inconspicuous ☐ Abdominal bloating ☐ Visible blood vessel ☐ Visible bowel loops ☐ Shiny ☐ Discoloured	Stomach appearance (put a X) ☐ Inconspicuous ☐ Abdominal bloating ☐ Visible blood vessel ☐ Visible bowel loops ☐ Shiny ☐ Discoloured	Stomach appearance (put a X) ☐ Inconspicuous ☐ Abdominal bloating ☐ Visible blood vessel ☐ Visible bowel loops ☐ Shiny ☐ Discoloured	Stomach appearance (put a X) ☐ Inconspicuous ☐ Abdominal bloating ☐ Visible blood vessel ☐ Visible bowel loops ☐ Shiny ☐ Discoloured
Stool since last meal? ☐ yes ☐ No	Stool since last meal? ☐ yes ☐ No	Stool since last meal? ☐ yes ☐ No	Stool since last meal? ☐ yes ☐ No
Stool amount (put a X) ☐1 ☐2 ☐3 ☐4	Stool amount (put a X) ☐1 ☐2 ☐3 ☐4	Stool amount (put a X) ☐1 ☐2 ☐3 ☐4	Stool amount (put a X) ☐1 ☐2 ☐3 ☐4
Blood in stool? ☐ yes ☐ No	Blood in stool? ☐ yes ☐ No	Blood in stool? ☐ yes ☐ No	Blood in stool? ☐ yes ☐ No
Stool consistency (put a X) ☐ A: watery ☐ B: Soft ☐ C: Formed ☐ D: Hard	Stool consistency (put a X) ☐ A: watery ☐ B: Soft ☐ C: Formed ☐ D: Hard	Stool consistency (put a X) ☐ A: watery ☐ B: Soft ☐ C: Formed ☐ D: Hard	Stool consistency (put a X) ☐ A: watery ☐ B: Soft ☐ C: Formed ☐ D: Hard
Stool colour (put a X) I II III IV V VI	Stool colour (put a X) I II III IV V VI	Stool colour (put a X) I II III IV V VI	Stool colour (put a X) I II III IV V VI
Medicine/vitamins pr OS? ☐ yes ☐ No If yes, which?	Medicine/vitamins pr OS? ☐ yes ☐ No If yes, which?	Medicine/vitamins pr OS? ☐ yes ☐ No If yes, which?	Medicine/vitamins pr OS? ☐ yes ☐ No If yes, which?
COMFORTneo Total score ______ Vurdering af distress ______	COMFORTneo Total score ______ Vurdering af distress ______	COMFORTneo Total score ______ Vurdering af distress ______	COMFORTneo Total score ______ Vurdering af distress ______
Gas restlessness ☐ yes ☐ No	Gas restlessness ☐ yes ☐ No	Gas restlessness ☐ yes ☐ No	Gas restlessness ☐ yes ☐ No

1

*MOM: Mother's own milk DHM: Donor human milk

Figure S1. Clips from the observation form.
